# Breeding Experience Might Be a Major Determinant of Breeding Probability in Long-Lived Species: The Case of the Greater Flamingo

**DOI:** 10.1371/journal.pone.0051016

**Published:** 2012-12-13

**Authors:** Roger Pradel, Rémi Choquet, Arnaud Béchet

**Affiliations:** 1 Roger Pradel Biostatistics and Population Biology Group, Centre d'Ecologie Fonctionnelle et Evolutive, Centre National de la Recherche Scientifique, Montpellier, France; 2 Rémi Choquet Biostatistics and Population Biology Group, Centre d'Ecologie Fonctionnelle et Evolutive, Centre National de la Recherche Scientifique, Montpellier, France; 3 Arnaud Béchet Population dynamics in response to human activities, Centre de Recherche de la Tour du Valat, Arles, France; Norwegian Polar Institute, Norway

## Abstract

The probability of breeding is known to increase with age early in life in many long-lived species. This increase may be due to experience accumulated through past breeding attempts. Recent methodological advances allowing accounting for unobserved breeding episodes, we analyzed the encounter histories of 14716 greater flamingos over 25 years to get a detailed picture of the interactions of age and experience. Survival did not improve with experience, seemingly ruling out the selection hypothesis. Breeding probability varied within three levels of experience : no breeding experience, 1 experience, 2+ experiences. We fitted models with and without among-individual differences in breeding probabilities by including or not an additive individual random effect. Including the individual random effect improved the model fit less than including experience but the best model retained both. However, because modeling individual heterogeneity by means of an additive static individual random effect is currently criticized and may not be appropriate, we discuss the results with and without random effect. Without random effect, breeding probability of inexperienced birds was always 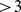 times lower than that of same age experienced birds, and breeding probability increased more with one additional experience than with one additional year of age. With random effects, the advantage of experience was unequivocal only after age 9 while in young having 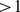 experience was penalizing. Another pattern, that breeding probability of birds with 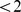 experiences dropped after some age (8 without random effect; up to 11 with it), may point to differences in the timing of reproductive senescence or to the existence of a sensitive period for acquiring behavioral skills. Overall, the role of experience appears strong in this long-lived species. We argue that overlooking the role of experience may hamper detection of trade-offs and assessment of individual heterogeneity. However, manipulative experiments are desirable to confirm our finding.

## Introduction

Breeding is at least a two-stage process: the decision to breed followed by the decision to allocate in the current reproduction [1]. Yet, studies of reproductive performance have often ignored non-breeding individuals [2], typically equating the average reproductive performance of breeders with that of similar individuals (e.g., same age) in the whole population [1]. The term ‘breeding success’ itself has come to mean ‘breeding success of breeders’. We will respect this tradition, reserving the term ‘reproductive success’ for the interesting parameter from an evolutionary point of view, namely the product of the probability of breeding by the breeding success of breeders. Despite being an important component of fitness for long-lived vertebrates, and one which has long retained the attention of ecologists [3], breeding probability [1], [4], synonymously breeding propensity [5], is much less studied than breeding success, e.g., [6]–[11]. Indeed, estimating breeding probabilities requires long-term studies of marked individuals [1] and an appropriate statistical framework (capture-recapture models, e.g., [12], [13].

In many long-lived species of vertebrates, probability of breeding tends to increase with age. This increase can be predicted on theoretical grounds. One line of reasoning is rooted in the optimal theory of life histories. This theory predicts that –except possibly in fast-growing populations (see [14], p. 218)– reproductive effort should increase with age. This is generally understood as an increase in reproductive effort when the expectation of future reproduction (residual reproductive value) declines with age [8], [15]. Hence, in long-lived species, breeding probability is expected to covary positively with age as a result of increasing reproductive effort, younger animals breed less frequently because they “restrain” from doing so (restraint or effort hypothesis). An alternative hypothesis proposes a lack of foraging and breeding efficiency in young, which progressively acquire the skills required to breed successfully. In this view, younger individuals would breed less frequently because they are incapable of doing so (constraint hypothesis [16]). Under this second hypothesis, experience might be more important than age per se in determining breeding probability (Table 1). Indeed, appropriate breeding skills (e.g., nest site defense, coordination of incubation duties between partners) are particularly likely to be acquired in the course of repeated breeding episodes. By contrast, the restraint hypothesis predicts no effect of experience. Even when there is no increase in breeding probability with age within an individual, among-individual differences may result in an observed pattern of age improvement at the population level [17]: selection eliminates poor performers first and the overall quality of the remaining pool of breeders increases mechanically (selection hypothesis, [2], [5]).

**Table 1:** Prediction of the role of experience in the increase of breeding probability with age.

Hypothesis	relevant time scale	mechanism	role of experience
pure restraint	evolutionary time	optimization of reproductive effort	no
pure constraint	lifetime	skill improvement	yes

Under a pure restraint hypothesis, breeding probability is hypothesized to increase as a response to the decline in residual reproductive value with age [61]. Under a pure constraint hypothesis, breeding probability increases through improved skills [2].

There is surprisingly few empirical tests examining, after controlling for age, whether individuals with previous breeding experience are more likely to breed, although the same question has been extensively examined for breeding success (reviews in [18]–[20]). Methological difficulties explain this different treatment. The few field studies examining the link between breeding experience and breeding probability rely on populations where capture probability is virtually equal to 1. In this way, breeding experience can be known exactly. Results are varied: no effect of breeding experience has been found in kittiwakes *Rissa tridactyla* [21], but experience has a positive effect on the probability of breeding in fulmar *Fulmarus glacialoides* [22] and blue petrel *Halobaena caerulea* [23]; in the first two studies, experience was measured as the number of years elapsed since the first breeding, which is different from the number of actual breeding experiences if the birds do not breed systematically; in the third study, age was unknown and not controlled for. In the endeavor to relate breeding probability to breeding experience, the obvious impediment is the incomplete breeding information inherent in most longitudinal studies. In most field studies of birds, observations are conducted at the breeding area during the breeding season. Non-observation of an animal can result either from the individual being missed by the observer, or the individual not being present. If a high level of philopatry can be assumed, non-breeding may be assimilated to an unobservable state. This common two-state approach, e.g., [24], [25] allows in particular testing the influence of age and of breeding allocation during the previous season on breeding probability [26]. However, it remains inadequate to examine the role of breeding experience. To account for the inherent uncertainty in the level of experience, capture-recapture models with a hidden Markov structure are required (multievent models [27]).

In this paper, we use the multievent framework to evaluate simultaneously the effects of age and experience on the breeding probability of the greater flamingos *Phoenicopterus roseus* breeding in the Camargue, southern France. Flamingos have a mate-switching rate 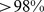 between consecutive breeding seasons [28]; hence, the effect of breeding experience may not be confounded with the effect of pair bond duration. Previous studies had suggested that breeding is a costly decision in this species especially in young age classes where, following a breeding episode, breeding probability (Tavecchia, unpublished manuscript) or immediate survival may be reduced [29]. Younger birds may refrain from expending maximal parental effort to forestall the associated risk of dying and, hence, losing future reproduction. On the other hand, because coordination of breeding duties in a mated pair is an important component of breeding outcome in flamingos, early breeding may pay off later in life. If breeding probability is solely shaped by restraint, we predicted breeding probability to increase with age independent of experience. However, if breeding probability is mainly shaped by constraint, we predicted breeding probability to increase with experience among birds of the same age. We examine this question on a large data set where we control for among-individual differences by means of an individual random effect [30], [31]. This approach is currently much debated by authors who contend that inter-individual differences in life histories should be represented by first-order Markov processes [32], [33]. We adopt here a pragmatic point of view and consider the individual random effect as the equivalent of a residual and do not try to interpret it. A restricted data set analysis using a measure of body condition at fledging as a covariate of breeding probability is presented in Appendix S3. This alternative approach to accounting for individual heterogeneity led to a similar conclusion as the analysis based on the full data set, namely that body condition while influential is less important than breeding experience as regards breeding probability.

## Materials and Methods

### Species, study area and data sets

Greater flamingos have bred in the saline lagoons of the Camargue, southern France, for centuries [34]. Since 1974, they have bred regularly on a 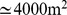 artificial island located in a complex of commercial salt pans where water levels are managed for salt production, except in 1996 when the birds settled on a nearby island following adverse conditions. Permissions for the observation and field studies were obtained from the company Salins. Since 1977, on average 12% (7–30%) of the chicks reared each year have been marked individually with PVC plastic rings engraved with alphanumerical codes which can be read through a telescope from a distance of up to 400 m. In 1983, a tower hide was erected near the breeding island, 70 m from the closest nest. Every spring throughout the breeding season, this hide is occupied by observers who record breeding activities of ringed birds. Flamingos are recorded as breeders when they are seen (i) at a nest with an egg, (ii) 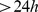 at the same position on the island (flag sticks allow precise positioning of each banded bird on the island), (iii) with a chick or (iv) sometimes only much later in the season feeding a chick in the crèche. Breeders that fail at the incubation stage may have a lower chance of being recorded as breeders as happens more often with young 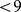 [35]. However, birds that fail early probably have then collected limited experience and it is considered extremely unlikely that they renest at another colony during the same breeding season [34]. In 1996, observation of breeding birds were performed from a distant dyke and using a floating hide. Since 1985, the ringing protocol includes biometrical measurement of chicks. In 2002, the monitoring was discontinued and restarted fully only 2 years later. The longest period when the data were collected continuously is thus 1977–2001.

### Multi-event modeling of breeding experience

**Figure 1:**
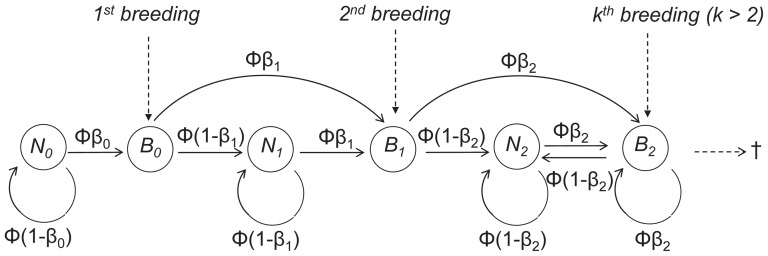
General pattern of transitions between breeding states. 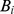
, breeder with 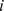 previous experiences and 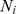, non-breeder with 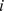 previous experiences for 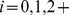. The transition probabilities are expressed in terms of 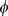, the probability of surviving to the next breeding season, and 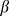, the probability, conditional on survival, of breeding the next season (the 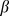
's will be age-dependent in practice).

Let us consider a bird of local experience 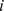, i.e. having bred exactly 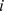 times before at the Camargue colony. This is an underestimation of total experience, acceptable for our purpose given the high level of fidelity to this colony ([36] estimate dispersal to another colony for the next breeding season to be 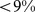 for first-time breeders and 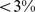 for experienced breeders) and conservative when it comes to detecting an effect of experience. For the model presentation, we limit the range of local experience (experience hereafter) to three levels: birds with no experience (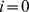), birds having bred once before (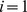) and birds with two or more previous breeding experiences (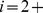). At each breeding occasion, a bird of experience 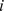 can either breed (state 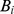) or not (state 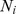). We denote 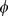 the apparent survival probability from one breeding season to the next and 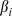 the breeding probability of a bird of experience level 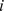. A non-breeding bird with no experience (state 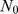) will remain so as long as it survives and does not breed with transitional probability 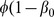). When it starts breeding, it joins the state 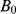 with probability 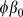. Because at this time it gains one year of experience, this state is only transitory. At the next occasion, it will be either a breeder with one previous experience (state 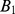) : probability 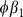; or a non-breeder with one previous experience (state 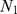) : probability 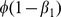. A bird in state 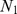 will remain in this state as long as it skips breeding : probability 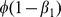. The acquisition of further breeding experiences (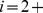) through subsequent breeding episodes follows the same pattern (Figure 1). When the colony is visited, there are just two possible events for a particular bird : either it is seen breeding (code 1) or not (code 0). We assume that a non-breeding bird is not present on the colony and thus cannot be observed and that, if the bird is breeding, it has a probability 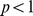 of being seen. After several seasons, the set of events make up a resighting history. The event on a particular occasion does not allow determination of the exact state of the bird. However, the estimation of the model parameters is possible in a probabilistic framework (for details on multievent modelling, see [27]). Details on the current model and its implementation may be found in Appendixes S1 and S2.

We analyzed the capture histories of 14716 flamingos marked as chicks from 1977 to 1997 and resighted until 2001. To focus on the breeding probabilities, we first decided on a survival and capture structure incorporating all the major known effects (see section 1.2 of Appendix S3 for a test of these effects). Building upon previous studies [29], [37], [38], we distinguished two age classes for survival probabilities, first-year and adult (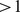 year-old). Survival probabilities were free to differ between the two age classes. They were maintained constant over all years except in the year separating breeding seasons 1984 and 1985 because in February 1985 a severe cold spell killed several thousand flamingos [34]. Resighting probabilities were time-specific and fixed to 0 for the first two years of life because sexual maturity is only reached at 3 years [39]. Breeding probabilities (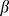) were calculated for each combination of age and experience with three levels of experience: inexperienced birds, birds that had bred once, birds that had bred at least two times. We also tried having 2 or 4 levels. Using the hybrid symbolic-numeric method developed by Rouan et al [40, section 3], we found that first-year survival could not be estimated separately from the breeding probabilities of inexperienced birds. Consequently, we decided to fix the first-year survival in normal years to a known value, which solved the identifiability problem. Based on a sex-ratio of 1/1 at birth and the sex-specific estimates of Balkız et al [41], we obtained a value of 0.763. With this value for normal years, first-year survival during the cold spell was estimated at 0.589, much lower than the sex-averaged estimate from Balkız: 0.697. However, because Balkız made her study with observations from the whole Mediterranean range of the species, her estimate unlike ours is unaffected by the dispersal of young from the Camargue, which may have been particularly high after the cold spell. Assuming that Balkız estimate is the true survival of young, losses would include roughly 16% dispersal of young. As a precaution, we also ran a model with a normal-year first-year survival of 0.632 corresponding to 17% emigration of young from the Camargue in normal years. Eventually, we introduced an additive random individual effect to control for individual heterogeneity in breeding probability and also tried to substitute it for experience. The models were implemented and run using program E-SURGE [42], which uses a quasi-Newton algorithm to minimize the deviance.

## Results

### Models without individual random effect

**Figure 2:**
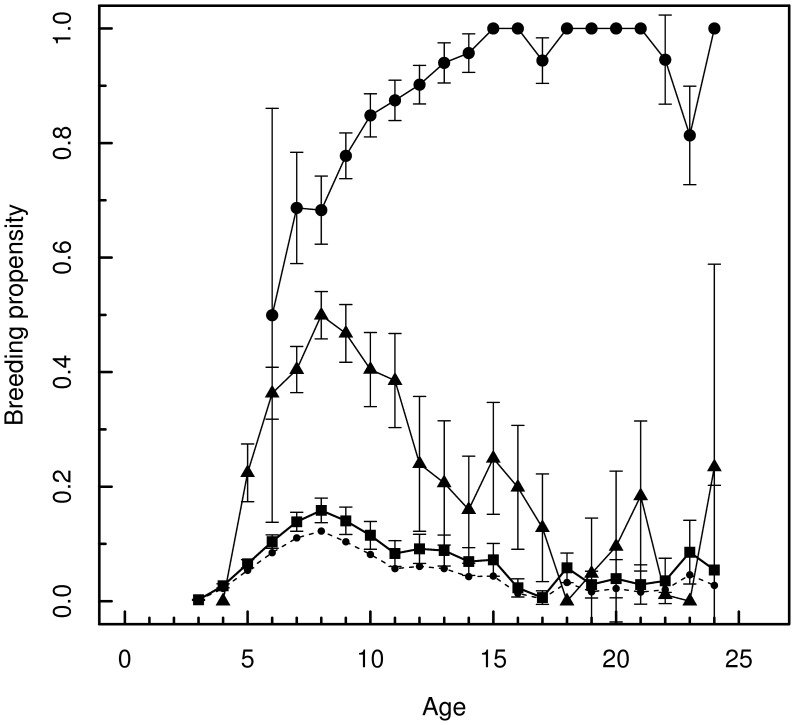
Breeding probability as a function of age and experience for greater flamingos breeding in the Camargue, south of France. full squares: no previous breeding episode; full triangles: one previous breeding episode; full circles: 2 or more previous breeding episodes. The curve for inexperienced individuals is that obtained with a normal-year first-year survival of 0.632; the dashed curve below is for a value of 0.763 of the same parameter corresponding to an absence of emigration (see text for details).

Figure 2 shows estimates of breeding probabilities of inexperienced individuals for two values of the first-year survival in normal years, the lowest curve (dashed line) corresponding to the assumption of no emigration in normal years. It should be noted that estimates of other parameters (including the breeding probabilities of birds with some experience) are completely insensitive to the amount of emigration assumed. Survival probabilities of adults were estimated at 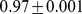 in normal years and at 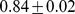 during the 1-year cold spell.

Breeding probability appeared clearly and strongly enhanced by experience (Figure 2). Breeding probabilities of birds with one or two previous breeding experiences present a right skewed shape with a peak at age 8. The breeding probability at age 8 of breeders with one previous experience (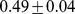) was 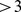 times higher than that of inexperienced breeders (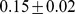). By contrast, breeding probabilities of multiple experienced flamingos increased continuously until they became systematic breeders (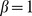) around age 15. Through model selection (Table 2), we checked that the model could not be simplified and in particular that experience and age were both important in determining the probability of breeding. Considering 3 levels of experience was better than 2 or 4 (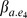 vs 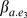: 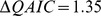). It is possible that more levels might have been necessary if we had used total experience instead of local experience.

**Table 2:** Modeling the effects of age and breeding experience on the breeding probability 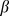 of greater flamingos marked as chicks from 1977 to 1997 and resighted as breeders until 2001 in the Camargue, southern France.

Model	Assumptions of main model or	Deviance	k	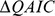
	– difference from main model			( 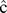 = 2.244)
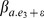	– additive individual terogeneity	53690.55	88	−47.93
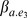	Breeding probability varies with age within 3 levels of previous breeding experience	53763.22	87	0
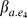	– one additional level of previous breeding experience	53700.24	105	1.35
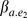	– only 2 levels of previous breeding experience	54047.69	67	86.76
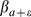	– individual heterogeneity instead of experience	54367.50	49	256.02
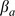	– no effect of experience	54707.50	47	340.79
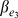	– no age effect	54926.11	29	402.20
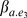 , 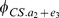 , 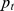	– additive effect of experience on survival	53755.37	89	−0.31
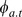 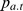	reference model for fit assessment	53883.71	439	757.69

Taking as a reference the fully age- and time-dependent survival and capture probabilities model [62], we calculated with program U-CARE [63] a variance inflation factor 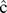 of 2.244 (section 1.1 of Appendix S3). In a first series of models, only the breeding probability 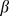 part varied: first-year apparent survival in normal years was fixed to 0.763; first-year apparent survival for the cold spell year 1984 was estimated separately; adult apparent survival was estimated separately for the cold spell year and normal years (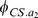); capture probability was time-dependent (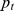). For the variable breeding probability part, experience was a factor with 2 levels (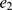): no experience, some previous experience; 3 levels (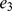): no experience, 1 previous experience, 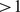 previous experiences; or 4 levels (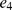): no experience, 1 previous experience, 2 previous experiences, 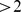 previous experiences; age (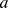) was a categorical variable. The notation ‘.’ means factorial model with main effects and interaction term. Our main model was 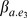 with 3 levels of experience interacting fully with age. Two models had an additive individual random effect (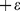). An additional model 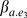, 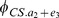, 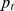 differed from the main model by an additive effect of experience on survival. 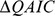 is the Quasi Akaike Information Criterion relative to the main model; k is the model rank.

### Models with individual random effect

**Figure 3:**
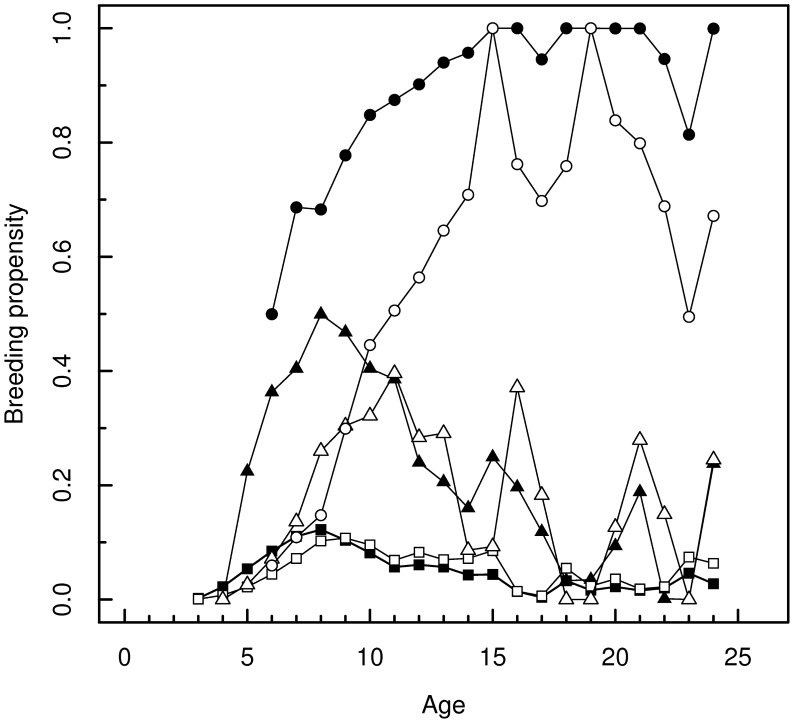
Change in breeding probability estimates in presence of an additive random individual effect. Comparison of the estimates of breeding probabilities under the main model 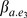 (thick line, full symbols) and those for an average individual under the additive random individual effect model 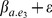 (thin line, empty symbols). Squares are for inexperienced individuals, triangles for individuals with one experience, and circles for individuals with 2+ experiences.

The individual random effect (model 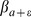) was by itself a poor substitute for experience (model 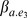). But the introduction of an additive individual random effect in addition to experience greatly improved the fit (model 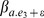). In presence of the random individual effect, the pattern of estimates of breeding probabilities over age and experience of an average individual is roughly similar to that obtained in the absence of the random effect (Figure 3) with some remarkable differences: breeding probabilities are lower for the young and remain so the longer, the more experienced the individual is. The peak for the inexperienced group and for the group with one experience is delayed to respectively 9 and 11 years. A major difference is that, up to age 9, those with 2+ experiences have a lower breeding probability than those with just 1 experience.

## Discussion

### Solutions to technical challenges

Studying the role of breeding experience on breeding probability from capture-recapture data presented two main challenges: breeding experience is not known exactly because of the imperfect detection of breeders and individual heterogeneity may obscure the pattern of variation of breeding probability with age if poor performers die earlier (selection hypothesis). The first point was solved by using capture-recapture models with a hidden Markov structure (multievent models). The second point could not be treated exactly as the nature of individual heterogeneity is imprecise. However, because the selection hypothesis implies that the average survival should increase with the level of experience as the average quality of survivors improves, we fitted a model with an additive effect of experience on survival. The fit was only marginally better (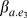, 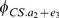, 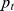 vs 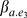, 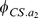, 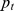: 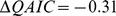) and the estimates were not consistent with the prediction (normal year adult survival: 0.982, 0.957, and 0.978 with increasing level of experience). Thus, the selection hypothesis does not seem to operate in our flamingo population. This does not preclude that individual heterogeneity is present in breeding propensity. We treated this aspect by introducing an additive individual random effect on breeding probability (capture-recapture mixed models [30]). This effect improved considerably the fit (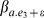 vs 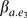: 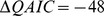). However, an individual random effect may reflect any kind of variation not explicitly incorporated in the model [33]. As an alternative, we considered using a measure of body condition at fledging available on a subset of the data (see Appendix S3). This measure was retained as a covariate of breeding probability but with opposite effects on each sex. Each approach has its shortcomings: one may be too general, the other too particular; one does not treat the two sexes separately, the other may not reflect differences in body condition later in life when it matters. Additionally for both, the particular relationship with breeding probability (additive effect on a logit scale) may not be appropriate. It remains that, in both approaches, experience came out as more important than the factor used to render individual heterogeneity. Because the pattern of variation of breeding probability with age and experience of an average individual in the restricted data set analysis was more similar to that in the model without individual heterogeneity, we will comment no more on this former analysis. In what follows, we comment preferentially on the traditional model (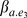, main model hereafter), signalling when relevant the differences with the mixed model (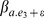).

### Importance of breeding experience

Results from our main model are consistent with past studies. The estimates of survival probabilities match those of Tavecchia et al [29]. Recapture probabilities do not differ from those found by Balkız et al [36] and the peak in breeding probability at age 8 was also found in recent analyzes not including the experience factor [41]. Breeding experience emerges from our analysis as the major factor determining breeding probability in our study population of Greater flamingos. Although both breeding experience and individual heterogeneity are present in the best model, it is breeding experience that explains the variations in breeding probability better (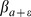 vs 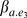: 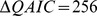). Our results rule out a pure restraint hypothesis which predicts no effect of experience (Table 1). On the other hand, our results are consistent with the constraint hypothesis. The decline in breeding probability in the least experienced past 8 years (9 according to the mixed model) evokes reproductive senescence. However, this comes early given the extended life expectancy of flamingos (40 y in the wild [29]) and concerns only the poorer breeders. There is no evidence of a parallel decline in survival, nor at the population level [29], nor in the corresponding category of breeding experience (model 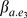, 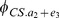, 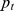). Alternatively, the decline could be due to an increasing inability to acquire needed skills past a sensitive period, e.g., displays involved in pair formation [43], in a manner reminiscent of the crystallization of song learning in songbirds [44] –although the mechanisms are likely to be quite different because the temporal scales are so different. The prominent role of experience is not well acknowledged in the theoretical literature on life history trade-offs. For instance, Stearns, in his reference book, does not even mention breeding experience when considering the benefits of early maturity [45, p 123–124]. Field biologists are aware of the potential influence of breeding experience on breeding probability [18] but, for lack of a practical solution, they have generally made simplifying assumptions like assuming that breeding was systematic after the first reproduction, e.g.,[46], but see [1], [4], [47] for an approach based on the robust design [48]. Yet, with the greater flamingo, we have a species where skipping reproduction is probably frequent. We estimate here that full breeding (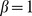) is not attained before age 15, and then only for the individuals with at least two previous breeding experiences (Figure 2). Given the large magnitude of the effect of breeding experience on breeding probability, one may wonder whether studies of generally weaker effects, like the cost of reproduction, have been affected in their conclusions when they ignored breeding experience. This should be particularly relevant in long-lived species. We review hereafter several common topics with which breeding experience may interfere, starting with the role of age.

### Experience vs age

Age had previously been shown to positively influence flamingo breeding behavior by ensuring better access either to breeding site [49] or to mate [28]. However, the confounding effect of experience could not be separately assessed. Here, we can examine the interplay of age and experience by picking a point on the lowest curve in Figure 2. This point corresponds to an inexperienced individual. An inexperienced individual with one more year of age lies at the next point on the same curve; an individual with one previous experience but the same age is found at the corresponding point of the intermediate curve. It appears that, from age 5 to age 17, it is always better to have one previous experience than one more year of age (age 4 is an apparent exception but see trade-offs below). According to the mixed model, the advantage of having one experience for an average individual is much weaker and it is only from age 7 that one experience is better than one year of age (Figure 3). After age 17, the data become too sparse to be reliably interpreted. For an individual with one previous experience (a point on the intermediate curve), it would generally be better to have one more year of experience than to be one year older. In fact, past age 7–8, age has essentially a negative effect on breeding probability in both 0–1 experience classes. However, here, the mixed model tells us a different story for ages 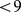 (Figure 3): if it is still good to have one experience, acquiring additional experiences causes a decline in breeding probability; it looks like in young too much breeding efforts is penalizing. On the other hand, improvement of breeding probability with age lasts longer, up to age 11. Several studies have demonstrated an increase of survival and breeding success from first-time breeders to experienced breeders [22], [23], [50], [51], but, to our knowledge, only another recent study has demonstrated the favorable cumulative effect of breeding experience on breeding probability [52].

### Skipping and reproductive trade-offs

Although trade-offs are phenomenons of major importance in the understanding of the evolution of life histories, in terms of magnitude they are second-order effects, except possibly in the young. Because of reproductive trade-offs, an individual which has bred one year may have a lower chance of breeding the next year. It is thus natural to study reproductive trade-offs in relation to breeding probability [25], [53]. It is often assumed that skipping is common among young breeders, then becomes less frequent and eventually there is an age of full breeding after which every individual still alive will breed systematically. The notion of a progressive acquisition of a regular breeding activity is well supported by our results because breeding probability increases with age until age 8 in the three experience groups. However, past age 8 (9 for inexperienced individuals and 11 for those with one experience according to the mixed model), a high probability of breeding holds only for individuals that have bred at least twice. The frequently held belief that there is an age of full breeding may stem from field observations that recognizable old individuals are breeding systematically year after year. However, our model predicts that there are old individuals almost never present on the breeding grounds because they breed only once or twice during their lives (see also sparrowhawk, [54, pp. 279–296]). These individuals may easily pass unnoticed during field studies of colonial birds where only breeders are observed.

The progressive increase of breeding probability in young of any species can be seen as an ontogenetic process by which individuals first acquire the physiological capacity to breed, then behavioural or social skills. This developmental process provides a rationale for a cost of early breeding. As soon as physiological maturation is achieved, reproduction becomes possible but it is inefficient and costly because some other skills have not been acquired or sufficiently perfected. An individual that nonetheless engages in breeding at an early age –as selection favors [55]– may thus have difficulties recovering and hence may have a reduced breeding probability the next year. A trade-off between successive breedings is difficult to examine from our analysis (Figure 2). Nonetheless, we note that flamingos having bred at age 3 do not breed at age 4 (9 birds were observed breeding at 3 years), although other still inexperienced individuals start breeding at 4 years. At this early age, the cost of reproduction appears thus to cancel an advantage of acquired experience. But this hierarchy of effects is rapidly reversed. At age 6, the individuals with two previous breeding episodes have necessarily bred at age 5 (because breeding at 3 precludes breeding at 4). Yet, they exhibit a higher breeding probability than individuals with just one experience of which at least some have not bred at 5. Thus, as early as 6, experience appears to override the potentially negative effect of a recent breeding. In contrast, the results of the mixed model plead for more durable effects of the cost of reproduction in the average individual (Figure 3). Breeding probability is generally lower than in the main model, particularly in young and in experienced individuals. But, above all, breeding a second time seems to be penalizing before age 9. This model thus suggests stronger and more lasting reproductive trade-offs. The difficulty of establishing the existence of trade-offs in the wild has long been put down to observational protocols [53] or to individual heterogeneity [56], but the role of experience is another possible reason.

### Implications in terms of life-history strategy

A turning point in the life history of the Camargue greater flamingos seems to lie somewhere around age 8 according to our main model, or later but before 11 according to the mixed model. Prior to that age, trade-offs have been found (reduced breeding probability: this study and Tavecchia, unpublished manuscript; reduced immediate survival [29]). Both costs are strongest in the youngest breeders and then diminish rapidly to become undetectable around age 6 according to the main model; they diminish more slowly and persist to age 9 according to the mixed model. These age-specific trade-offs suggest breeding at the earliest possible age is probably not optimal because the associated costs are then too high to be compensated for by expected higher future breeding outcomes as a result of experience. Yet, a pivotal age must exist where the long-term gains of experience balance the short-term cost of reproduction. Apart from the greater flamingos, there is accumulating evidence that trade-offs are especially strong early in life when reproduction seems to be costly, particularly for first time breeders [21], [23], [51], [57], [58]. On the other hand, the generality of our result on the importance of experience in breeding probability must be confirmed. If experience is confirmed as a major factor acting on breeding probability, the way we understand life history strategy may have to be changed. Experience and cost of reproduction would then appear as the two dominant opposite forces vying to determine the optimal age of first breeding.

## Conclusions

Among the natural candidate factors potentially determining the breeding probability of an individual, breeding experience had rarely been assessed for lack of suitable methods. Using novel statistical tools for capture-recapture data, we have developed a simple and universal model applicable to any species where individuals are recognizable (simple generalizations like observations of non-breeders or inclusion of several sites to approach more closely total experience should be straightforward). We have found that breeding experience is a major factor in flamingos. We have then drawn the consequences of our finding in relation to major topics of the life history theory. It appears that the difficult and yet central point in studies of breeding probability is to correctly model individual heterogeneity. Progress in statistical models could help in this respect. However, we believe that it is only through manipulative experiments that the exact part of each factor can be assessed. A possible approach could be to prevent a random sample of young individuals from breeding for several years, possibly through hormonal manipulation [59], [60], and then compare their breeding probability to that of control individuals.

## Supporting Information

Appendix S1 Multi-event modeling of breeding experience.
(PDF)

Appendix S2 Implementation of the generic model in E-SURGE.
(PDF)

Appendix S3 Results of data analysis.
(PDF)
