# Supplementary material for: Breeding Experience Might Be a Major Determinant of Breeding Probability in Long-Lived Species: The Case of the Greater Flamingo
Source: PLoS One. 2012 Dec 13;7(12):e51016. doi: 10.1371/journal.pone.0051016 (PMC3521775; doi:10.1371/journal.pone.0051016)
Supplement: Appendix S1 — Multi-event modeling of breeding experience. (PDF) [file pone.0051016.s001.pdf]

Multi-event modeling of breeding experience

The multievent framework [1] distinguishes the *events*, coded in the capture histories, from the *states* which are not directly observed. When studying experience, there are several states to consider while there are only 2 events.

The events are:

- 0, 'not observed',
- 1, 'observed'.

The states retained are:

- NB_0, non-breeder with no previous experience at the onset of the season,
- B_0, breeder with no previous experience at the onset of the season,
- NB_1, non-breeder with one previous experience at the onset of the season,
- B_1, breeder with one previous experience at the onset of the season,
- NB_2, non-breeder with > 1 previous experience at the onset of the season,
- B_2, breeder with > 1 previous experience at the onset of the season.

A multievent model has three kinds of parameters : the *initial state probabilities*, the *transition probabilities*, and the *event probabilities* (conditional on the underlying state). As every individual in our data set was ringed as a chick, all initial state probabilities are trivially 1 for the state NB_0 and this kind of parameters is not used here.

The transitions are best presented in stochastic matrix form with departure states in rows and arrival states in columns (see also Figure 1 in main text). ϕ is the survival probability, β the breeding probability. β is indexed according to the level of previous experience: 0 no previous experience, 1 one previous experience, and 2 two or more previous experiences.

$$\begin{array}{c} NB_0 \\ B_0 \\ NB_1 \\ B_1 \\ NB_2 \\ B_2 \\ dead \end{array} \overset{\begin{array}{ccccccc} NB_0 & B_0 & NB_1 & B_1 & NB_2 & B_2 & dead \end{array}}{\begin{pmatrix} \phi(1-\beta_0) & \phi\beta_0 & 0 & 0 & 0 & 0 & 1-\phi \\ 0 & 0 & \phi(1-\beta_1) & \phi\beta_1 & 0 & 0 & 1-\phi \\ 0 & 0 & \phi(1-\beta_1) & \phi\beta_1 & 0 & 0 & 1-\phi \\ 0 & 0 & 0 & 0 & \phi(1-\beta_2) & \phi\beta_2 & 1-\phi \\ 0 & 0 & 0 & 0 & \phi(1-\beta_2) & \phi\beta_2 & 1-\phi \\ 0 & 0 & 0 & 0 & \phi(1-\beta_2) & \phi\beta_2 & 1-\phi \\ 0 & 0 & 0 & 0 & 0 & 0 & 1 \end{pmatrix}}$$

Note that most transitions are impossible, that the last state 'dead' can be reached from any live state with probability $1 - \phi$ and that once in this state an individual will remain in it forever (transition from 'dead' to 'dead' is 1 : last row, last column). This basic model can be particularized, for instance to allow for a first-year survival lower than survival in later years or to introduce time-dependency on breeding probability.

The event probabilities relate the observations coded in the capture histories to the underlying states. Here, we assume that non-breeders, except chicks at the time of ringing (state NB_0), cannot be observed (event '0' with probability 1) and that breeders, whatever their experience, share the same probability

p to be observed. A matrix form with the conditioning state in row and the event in column is again a particularly handy presentation.

$$\begin{array}{c} \\ NB_0 \\ B_0 \\ NB_1 \\ B_1 \\ NB_2 \\ B_2 \\ dead \end{array} \begin{array}{c} \begin{array}{cc} \text{'0'} & \text{'1'} \end{array} \\ \begin{pmatrix} 1 & 0 \\ 1-p & p \\ 1 & 0 \\ 1-p & p \\ 1 & 0 \\ 1-p & p \\ 1 & 0 \end{pmatrix} \end{array}$$

References

1. Pradel R (2005) Multievent: an extension of multistate capture-recapture models to uncertain states. Biometrics 61: 442-447.
