# Supplementary material for: Breeding Experience Might Be a Major Determinant of Breeding Probability in Long-Lived Species: The Case of the Greater Flamingo
Source: PLoS One. 2012 Dec 13;7(12):e51016. doi: 10.1371/journal.pone.0051016 (PMC3521775; doi:10.1371/journal.pone.0051016)
Supplement: Appendix S2 — Implementation of the generic model in E-SURGE. (PDF) [file pone.0051016.s002.pdf]

Implementation of the generic model in program E-SURGE

We present here how to implement the above model using program E-SURGE. The specifics of the model have been kept as general as possible in order to be applicable to data sets other than the one we used in the article. For instance, we have not included the effect of one very cold year on survival. However, some details, which should be easy to adapt, relate to the flamingo example. For instance, no breeding is possible until age 3. We intend this as a template for other studies.
E-SURGE can be downloaded at http://www.cefe.cnrs.fr/biom/logiciels.htm. It can read in data in a no-frill MARK format: no comments, no tabulations, no letters in the capture histories (only digits); or alternatively in BIOMECO format (see the associated manual for a description).

After opening a new session and reading in the data (2 first items of the bar menu), the number of states, 7, has to be specified through the 'Modify' button.

It is now possible to go through the 4 steps represented by the coloured buttons at the bottom left.

1 specifying the patterns (tool GEPAT)

This stage roughs out the model by specifying the probabilities that will not be used (impossible initial states, transitions, or events)– code '-' (minus sign) –and those that will be calculated from others (because they are the last of a set of exclusive complementary options)– code '*'.

Initial States:

$$\begin{bmatrix} \star & - & - & - & - & - \end{bmatrix}$$

We decompose the transition in two steps: first survival, then the decision to breed.

Survival Step:

$$\begin{bmatrix} \phi & - & - & - & - & - & \star \\ - & \phi & - & - & - & - & \star \\ - & - & \phi & - & - & - & \star \\ - & - & - & \phi & - & - & \star \\ - & - & - & - & \phi & - & \star \\ - & - & - & - & - & \phi & \star \\ - & - & - & - & - & - & \star \end{bmatrix}$$

Breeding Step:

$$\begin{bmatrix} \star & \beta & - & - & - & - & - \\ - & - & \star & \beta & - & - & - \\ - & - & \star & \beta & - & - & - \\ - & - & - & - & \star & \beta & - \\ - & - & - & - & \star & \beta & - \\ - & - & - & - & \star & \beta & - \\ - & - & - & - & - & - & \star \end{bmatrix}$$

Event probabilities:

$$\begin{bmatrix} \star & p \\ \star & p \\ \star & - \\ \star & p \\ \star & - \\ \star & p \\ \star & - \end{bmatrix}$$

The p on the first row (state NB_0) is needed because chicks are observed. We will clarify this point in the next step, GEMACO [1]. (Note that GEPAT only displays greek letters; this is not a problem.)

2 specifying the effects (tool GEMACO)

Initial States: there is nothing to do here

Survival step:

$$a(1, 2:100)$$

This assumes that survival is constant over age past the first year of life. The last number (100 here) must exceed the number of time steps.

Breeding step:

$$a(3\_25).to(2) + a(4\_25).to(4) + a(5\_25).to(6) + others$$

The breeding probabilities of inexperienced individuals ($to(2)$) are estimated from age 3 to age 25 (maximum age observable in the flamingo data set), those for individuals with one previous breeding experience ($to(4)$) from age 4 to 25, those for more experienced individuals ($to(6)$) from age 5 to 25. No individual breeds prior to age 3. The unspecified breeding probabilities, which should all be 0, are gathered together as a last breeding probability by the keyword '*others*'.

Event probabilities:

$$firste + nexte.[f(2\,4\,6)].t + others$$

Here, we distinghish the initial encounter probability (*firste*) from the subsequent probabilities of encountering breeders ($f(2\,4\,6)$) which are allowed to vary over years (t). The remainder are the subsequent probabilities of encountering non-breeders (*others*).

3 setting some parameter values (tool IVFV)

The last transition corresponds, among others, to the breeding probability prior to age 3 and must be set to 0 (by entering the value 0 on the right and checking the box next to it). The first event is the probability of encounter at the time of marking. It must be set to 1. The last event is the probability of encountering adult non-breeders. It has to be set to 0.

4 RUN

The model can now be run.

References

1. Choquet R (2008) Automatic generation of multistate capture-recapture models. Canadian Journal of Statistics 36: 43–57.
