# Supplementary material for: Breeding Experience Might Be a Major Determinant of Breeding Probability in Long-Lived Species: The Case of the Greater Flamingo
Source: PLoS One. 2012 Dec 13;7(12):e51016. doi: 10.1371/journal.pone.0051016 (PMC3521775; doi:10.1371/journal.pone.0051016)
Supplement: Appendix S3 — Results of data analysis. (PDF) [file pone.0051016.s003.pdf]

# Results of data analysis

# 1 Complete data set: years 1977-2001

## 1.1 Assessment of goodness-of-fit

There is no goodness-of-fit test available for the breeding probability models. We examined the fit of the fully time- and age-dependent survival and capture model [1]. This test is obtained by running the test of the Cormack-Jolly-Seber model separately on each cohort and summing over cohorts (Table 1). Calculations were made with program U-CARE [2]. This led to a variance inflation factor $\hat{c}$ of 2.244 (1355.43/604) for the Pollock model. This value is conservative when applied to a model that performs better than the Pollock model. We used it for our analysis after introducing the Pollock model for comparison among the models fitted.

## 1.2 Additional models

We checked with two models that we could not rid ourselves of the cold spell effect on survival nor of the temporal variability of capture rates (Table 2).

# 2 Detailed data set: years 1985-2001

## 2.1 Goodness-of-fit

Same procedure as for the complete data set (Table 3). Variance inflation factor $\hat{c}$ estimated at 1.625 (247.07/152) for the Pollock model.

## 2.2 estimates from best model

With the reduced data set, more information was available about individuals. Also, during the restricted period, no major climatic events took place so that survival could be considered constant within each class of individuals. We took advantage of this new context to examine the influence of sex and of individual differences on breeding probabilities and to refine the modelisation of survival. The age and sex survival structure was matched exactly to that of [3], who has found sex differences and a second-year survival still somewhat lower than the adult survival. The cost of first reproduction in females (notation 1rf), first detected by [4], was introduced as a smoothed function of age (logit(age) is a linear function of log(age) that cancels at 6, see Table 4 below). Individual differences were introduced through the use of the residuals of the log-log regression of weight on tarsus length at ringing as a covariate of breeding probability. The best model retained is $\phi_{a3.s+1rf}$ $\beta_{a_2.e+BC.s}$ $p_{t+s}$ i.e. sex- and age-specific survival (with 3 age classes) with an age-specific cost of first reproduction in females, capture rates varying over time in parallel (on a logit scale) in the two sexes, and breeding probabilities age- and experience-dependent and affected by the body condition differently in females and males (Figure 1 and (Figure 2). A sex-specific effect of body condition on survival was also tested but not retained (see next section).

## 2.3 effect of body condition on survival

To test for individual differences in survivorship after accounting for the cost of the first reproduction in females, body condition was introduced as a covariate of survival for males and females separately in the best model $\phi_{a3.s+1rf}$ $\beta_{a_2.e+BC.s}$ $p_{t+s}$ (see above section). The effect was not significant (Table 7).

# References


1. Pollock KH (1981) Capture-recapture models allowing for age dependant survival and capture rates. Biometrics 37: 521-529.

2. Choquet R, Lebreton J, Gimenez O, Reboulet A, Pradel R (2009) U-CARE: Utilities for performing goodness of fit tests and manipulating capture–recapture data. Ecography 32: 1071–1074.

3. Balkız Ö (2006) Dynamique de la métapopulation de flamants roses en Méditerranée: implications pour la conservation. Ph.D. thesis, Université Montpellier II.

4. Tavecchia G, Pradel R, Boy V, Johnson AR, Cezilly F (2001) Sex- and age-related variation in survival and cost of first reproduction in greater flamingos. Ecology 82: 165-174.


# Figures

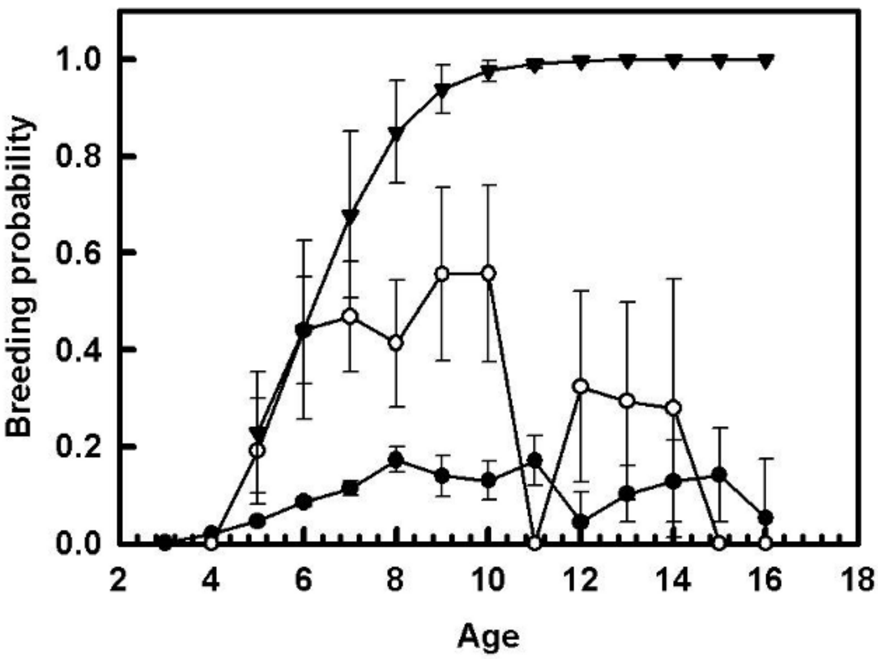


**Figure 1. Breeding probability as a function of age and experience for greater flamingos breeding in the Camargue, south of France, estimated from the reduced data set (1985-2001).** Plain circle: no previous breeding episode; empty circle: one previous breeding episode; triangle: 2 or more previous breeding episodes. These values are for an average individual in terms of body condition: value 0 of the covariate.

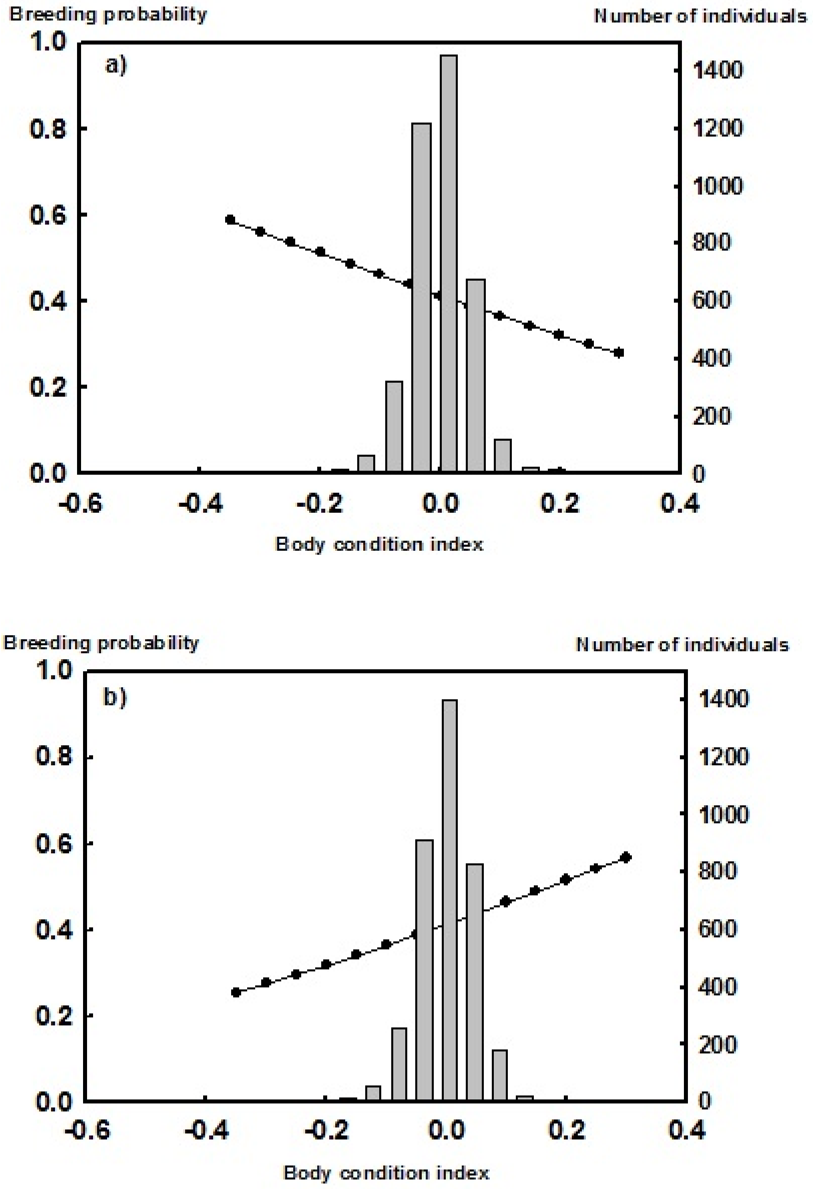


**Figure 2. Influence of body condition on breeding probability.** a) females, b) males. Positive values of the index correspond to larger than average individuals. The histogram shows the actual distribution of body condition in the population for each sex. The values of the curves are for 8-year-old individuals with one previous breeding experience.

# Tables

**Table 1. Goodness-of-fit tests of the time-dependent survival and capture Cormack-Jolly-Seber model by cohort.**

| Cohort | $\chi^2$ | degrees of freedom |
|---|---|---|
| 1977 | 165.95 | 64 |
| 1978 | 199.48 | 65 |
| 1979 | 169.96 | 67 |
| 1980 | 134.80 | 55 |
| 1981 | 174.96 | 60 |
| 1982 | 122.35 | 52 |
| 1983 | 78.69 | 48 |
| 1984 | 58.65 | 39 |
| 1985 | 80.05 | 39 |
| 1986 | 42.09 | 28 |
| 1987 | 40.08 | 23 |
| 1988 | 36.52 | 21 |
| 1989 | 14.53 | 12 |
| 1990 | 8.94 | 9 |
| 1991 | 7.51 | 9 |
| 1992 | 14.43 | 7 |
| 1993 | 6.43 | 5 |
| 1994 | 0 | 1 |
| 1995-1998 | 0 | 0 |
| Total | 1355.43 | 604 |

**Table 2. Some additional models in the analysis of the effects of age and breeding experience on the breeding probability $\beta$ of greater flamingos marked as chicks from 1977 to 1997 and resighted as breeders in the Camargue, southern France, until 2001.**

| Model | Best or Reference model or – hypothesis tested | Deviance | k | $\Delta$QAIC ($\hat{c}$=2.244) |
|---|---|---|---|---|
| $\beta_{a.e}$, $\phi_{CS.a2}$, $p_t$ | Best model | 53763.22 | 87 | 0 |
| $\beta_{a.e}$, $\phi_{a2}$, $p_t$ | – no effect of cold spell on survival | 53803.83 | 85 | 14.60 |
| $\beta_{a.e}$, $\phi_{CS.a2}$, $p$ | – capture rate constant | 55295.91 | 66 | 641.50 |
| $\phi_{a.t}$ $p_{a.t}$ | Reference Pollock model | 53883.71 | 439 | 757.69 |

Notations are as in Table 2 of the main document except for $a2$ which means two age-classes, the first one corresponding to the first year of life. CS stands for 'cold spell'. In the models with a cold spell effect, first-year survival in normal years is fixed to 0.763, the sex-averaged value of [3] estimates.

**Table 3. Goodness-of-fit tests of the time-dependent survival and capture Cormack-Jolly-Seber model by cohort for the 1985-2001 dataset.**

| Cohort | $\chi^2$ | degrees of freedom |
|---|---|---|
| 1985 | 78.04 | 38 |
| 1986 | 41.59 | 28 |
| 1987 | 38.74 | 22 |
| 1988 | 36.52 | 21 |
| 1989 | 14.53 | 12 |
| 1990 | 9.41 | 9 |
| 1991 | 7.51 | 9 |
| 1992 | 14.43 | 7 |
| 1993 | 6.30 | 5 |
| 1994 | 0 | 1 |
| 1995-1998 | 0 | 0 |
| Total | 247.07 | 152 |

**Table 4. Survival estimates from the best model fitted for the 1985-2001 dataset**

| | | 95% C.I. | | |
|---|---|---|---|---|
| parameter | estimate | lower boundary | higher boundary | s.e. |
| female 1st year | 0.779 | fixed parameter | | - |
| male 1st year | 0.747 | fixed parameter | | - |
| female 2nd year | 0.944 | fixed parameter | | - |
| male 2nd year | 0.921 | fixed parameter | | - |
| female 3+ year | 0.970 | 0.951 | 0.981 | 0.007 |
| male 2+ year | 0.957 | 0.943 | 0.968 | 0.006 |
| first breeding 3-year-old female | 0.651 | 0.492 | 0.782 | 0.076 |
| first breeding 4-year-old female | 0.858 | 0.774 | 0.915 | 0.035 |
| first breeding 5-year-old female | 0.938 | 0.900 | 0.962 | 0.015 |

**Table 5. Breeding estimates from the best model for the detailed dataset: years 1985-2001.**

| # previous breeding episodes | age | point estimate | 95% C.I. lower boundary | 95% C.I. higher boundary | s.e. |
|---|---|---|---|---|---|
| 0 | 3 | 0.002 | 0.001 | 0.005 | 0.001 |
| 0 | 4 | 0.020 | 0.015 | 0.027 | 0.003 |
| 0 | 5 | 0.046 | 0.037 | 0.058 | 0.005 |
| 0 | 6 | 0.085 | 0.069 | 0.105 | 0.009 |
| 0 | 7 | 0.114 | 0.088 | 0.145 | 0.014 |
| 0 | 8 | 0.173 | 0.128 | 0.229 | 0.026 |
| 0 | 9 | 0.139 | 0.074 | 0.246 | 0.043 |
| 0 | 10 | 0.130 | 0.069 | 0.232 | 0.041 |
| 0 | 11 | 0.171 | 0.091 | 0.297 | 0.052 |
| 0 | 12 | 0.044 | 0.002 | 0.469 | 0.063 |
| 0 | 13 | 0.102 | 0.033 | 0.279 | 0.057 |
| 0 | 14 | 0.128 | 0.032 | 0.394 | 0.085 |
| 0 | 15 | 0.141 | 0.033 | 0.442 | 0.097 |
| 0 | 16 | 0.053 | 0.000 | 0.872 | 0.123 |
| 1 | 4 | 0.000 | 0.000 | 0.000 | 0.000 |
| 1 | 5 | 0.191 | 0.056 | 0.486 | 0.109 |
| 1 | 6 | 0.440 | 0.246 | 0.654 | 0.110 |
| 1 | 7 | 0.468 | 0.265 | 0.683 | 0.113 |
| 1 | 8 | 0.413 | 0.196 | 0.671 | 0.131 |
| 1 | 9 | 0.556 | 0.234 | 0.837 | 0.178 |
| 1 | 10 | 0.557 | 0.228 | 0.843 | 0.182 |
| 1 | 11 | 0.000 | 0.000 | 0.000 | 0.000 |
| 1 | 12 | 0.323 | 0.075 | 0.737 | 0.197 |
| 1 | 13 | 0.294 | 0.057 | 0.741 | 0.204 |
| 1 | 14 | 0.280 | 0.028 | 0.838 | 0.266 |
| 1 | 15 | 0.000 | 0.000 | 0.000 | 0.000 |
| 1 | 16 | 0.000 | 0.000 | 0.000 | 0.000 |
| 2+ | 5 | 0.229 | 0.069 | 0.543 | 0.125 |
| 2+ | 6 | 0.442 | 0.154 | 0.775 | 0.185 |
| 2+ | 7 | 0.679 | 0.310 | 0.909 | 0.172 |
| 2+ | 8 | 0.849 | 0.525 | 0.966 | 0.106 |
| 2+ | 9 | 0.938 | 0.731 | 0.988 | 0.051 |
| 2+ | 10 | 0.976 | 0.870 | 0.996 | 0.022 |
| 2+ | 11 | 0.991 | 0.943 | 0.999 | 0.009 |
| 2+ | 12 | 0.997 | 0.976 | 1.000 | 0.003 |
| 2+ | 13 | 0.999 | 0.990 | 1.000 | 0.001 |
| 2+ | 14 | 1.000 | 0.996 | 1.000 | 0.001 |
| 2+ | 15 | 1.000 | 0.998 | 1.000 | 0.000 |
| 2+ | 16 | 1.000 | 0.999 | 1.000 | 0.000 |

**Table 6. Capture estimates. Confidence intervals are not available for the parameters estimated on the boundary. No individuals were at risk of capture prior to 1988.**

| | | 95% C.I. | | |
|---|---|---|---|---|
| year | estimate | lower boundary | higher boundary | s.e. |
| | | females | | |
| 1988 | 0.000 | - | - | - |
| 1989 | 0.000 | - | - | - |
| 1990 | 0.496 | 0.247 | 0.747 | 0.140 |
| 1991 | 1.000 | - | - | - |
| 1992 | 0.405 | 0.296 | 0.525 | 0.059 |
| 1993 | 0.254 | 0.191 | 0.329 | 0.035 |
| 1994 | 0.314 | 0.252 | 0.384 | 0.034 |
| 1995 | 0.374 | 0.314 | 0.438 | 0.032 |
| 1996 | 0.241 | 0.200 | 0.287 | 0.022 |
| 1997 | 0.305 | 0.260 | 0.354 | 0.024 |
| 1998 | 0.418 | 0.367 | 0.471 | 0.027 |
| 1999 | 0.179 | 0.152 | 0.209 | 0.015 |
| 2000 | 0.373 | 0.331 | 0.417 | 0.022 |
| 2001 | 0.331 | 0.293 | 0.371 | 0.020 |
| | | males | | |
| 1988 | 0.000 | - | - | - |
| 1989 | 0.000 | - | - | - |
| 1990 | 0.569 | 0.304 | 0.800 | 0.138 |
| 1991 | 1.000 | - | - | - |
| 1992 | 0.477 | 0.360 | 0.597 | 0.062 |
| 1993 | 0.313 | 0.239 | 0.398 | 0.041 |
| 1994 | 0.381 | 0.310 | 0.457 | 0.038 |
| 1995 | 0.445 | 0.379 | 0.512 | 0.034 |
| 1996 | 0.298 | 0.250 | 0.352 | 0.026 |
| 1997 | 0.370 | 0.318 | 0.425 | 0.027 |
| 1998 | 0.490 | 0.434 | 0.547 | 0.029 |
| 1999 | 0.226 | 0.193 | 0.263 | 0.018 |
| 2000 | 0.443 | 0.397 | 0.491 | 0.024 |
| 2001 | 0.399 | 0.355 | 0.443 | 0.023 |

**Table 7. Test of an effect of body condition on survival assessed from the best model $\phi_{a3.s+1rf}$ $\beta_{a_2.e+BC.s}$ $p_{t+s}$. Only the survival part is shown below as the remainder is unchanged.**

| Model | Best model or – difference from best model | Deviance | k | ΔQAIC ($\hat{c}$=1.625) |
|---|---|---|---|---|
| $\phi_{a3.s+1rf}$ | Best model | 17575.27 | 49 | 0 |
| $\phi_{a3.s+1rf+BC.s}$ | – effect of body condition on survival assessed separately on males and females | 17574.79 | 51 | 3.70 |
